# Pediatric Heterotopic Gastric Mucosa of the Cervical Esophagus (Inlet Patch): Case Series with Clinical, Endoscopic, and Histopathological Correlation

**DOI:** 10.3390/children12060752

**Published:** 2025-06-10

**Authors:** Javier Arredondo Montero, Samuel Sáez Álvarez, Andrea Herreras Martínez, Ana Fernández-García, Cristina Iglesias Blázquez

**Affiliations:** 1 Pediatric Surgery Department, Complejo Asistencial Universitario de León, 24008 León, Castilla y León, Spain; 2 Pathology Department, Complejo Asistencial Universitario de León, 24008 León, Castilla y León, Spain; 3 Pediatrics Department, Complejo Asistencial Universitario de León, 24008 León, Castilla y León, Spain

**Keywords:** Inlet patch, pediatric, upper gastrointestinal endoscopy, esophagus, gastric heterotopia, histology, inflammation, gastritis, *Helicobacter pylori*

## Abstract

**Introduction:** Inlet patch (IP) is a congenital anomaly characterized by gastric heterotopia in the cervical esophagus. While extensively described in adults, it remains poorl characterized in pediatric populations. **Material and Methods:** This retrospective, single-center study included all pediatric patients (0–14 years) diagnosed with IP between 2018 and 2025. Sociodemographic and clinical data were collected. A blinded pathologist assessed the presence and severity of inflammation within the IP. **Results:** Nine patients (median age, 12 years; range, 6–14 years) were included, with 78% beingmale. Cervical esophageal symptoms were identified in 67%, primarily dysphagia and gastroesophageal reflux disease-related complaints, although concomitant conditions such as eosinophilic esophagitis were frequently present. Three patients had symptoms potentially attributable to IP (33%). Endoscopic examination revealed characteristic well-demarcated salmon-red plaques in all patients, with multiple lesions observed in three cases. Histology confirmed gastric heterotopia with varying degrees of chronic inflammation in all cases. A potential association was observed between the severity of gastritis in the stomach, the severity of inflammation in the IP, and the presence of *H. pylori*, with 75% of patients with moderate-to-severe IP inflammation also exhibiting gastric *H. pylori*-associated gastritis. All patients except one received proton pump inhibitors, and symptoms improved in all cases. **Conclusions:** A thorough and targeted examination of the cervical esophagus significantly increased IP detection at our center, with most cases (89%) being diagnosed in the last 12 months. While mostly asymptomatic and incidental, IP can be symptomatic. In this case, series, we found a possible association between the severity of inflammation in the IP, the severity of gastritis, and the presence of *H. pylori*. Further studies are needed to define the clinical significance of pediatric IP and optimal management.

## 1. Introduction

Inlet patch (IP) is an anomaly characterized by gastric heterotopia in the cervical esophagus [1,2,3]. It is considered an infrequent pathology, especially in pediatric populations, although the literature regarding its prevalence is inconsistent [1,2,3,4,5,6,7].

IP etiology is presumed to be congenital, resulting from abnormal epithelial development during embryogenesis [8,9]. However, alternative hypotheses—such as an acquired origin secondary to metaplastic changes in the squamous mucosa caused by chronic acid injury—have also been proposed [7].

Clinically, IP is most often an asymptomatic lesion, incidentally diagnosed during upper gastrointestinal endoscopy (UGIE) performed for unrelated reasons. It should be noted, however, that its identification can be challenging due to its proximal location within the esophagus [1,2,3,4,5,6,7]. Nevertheless, a wide range of IP-related symptoms has been described, including dyspepsia, dysphagia, esophageal strictures or membranes, gastroesophageal reflux, bleeding, and even respiratory manifestations such as chronic bronchitis or laryngospasm [9,10,11,12,13].

From an endoscopic perspective, it is a distinctive lesion, appearing as a well-demarcated pink or salmon-colored patch. It is typically located in the upper third of the esophagus, just below the upper esophageal sphincter (UES). However, it has also been described, albeit exceptionally, in other locations, such as the mid-esophagus [14].

Although there is general agreement that incidentally detected IP should not be treated in asymptomatic patients, evidence on the management of symptomatic IP remains scarce. Proton pump inhibitors (PPIs) are considered the first-line treatment [1,2,3], as they are effective in most cases, while endoscopic therapies, such as argon plasma coagulation, are reserved for refractory cases [15]. This study aims to characterize pediatric cases diagnosed with IP at our center from 2018 to 2025.

## 2. Materials and Methods

This retrospective, single-center study included all pediatric patients (0–14 years) diagnosed with IP at our institution between 2018 and 2025. Two researchers collected and anonymized the patients’ sociodemographic and clinical data. A blinded pathologist evaluated all biopsies and classified the degree of IP inflammation using a qualitative scale: absence of inflammation, mild chronic inflammation, moderate to severe chronic inflammation, and acute inflammation. A descriptive analysis was performed. The study was approved by our Institutional Review Board (code 24227). Written informed consent was obtained from all legal guardians of all patients before inclusion. The study was conducted following the principles outlined in the Declaration of Helsinki (2013 statement).

## 3. Results

Nine patients were included, with a median age at diagnosis of 12 years (range: 6–14). Seven were male (78%), and two were female (22%).

Regarding medical history, four patients (44%) had a prior diagnosis of eosinophilic esophagitis (EoE). Among them, one also had a concomitant diagnosis of celiac disease (CD). In contrast, another had a serological and genetic profile compatible with CD but did not meet the ESPGHAN 2012 criteria, nor had histopathological confirmation of CD. Additionally, one patient in the series had insulin-resistant type 1 diabetes mellitus, and another had a previous diagnosis of gastroesophageal reflux (GER) secondary to *H. pylori* infection. Two patients had no relevant medical history.

**Figure 1:**
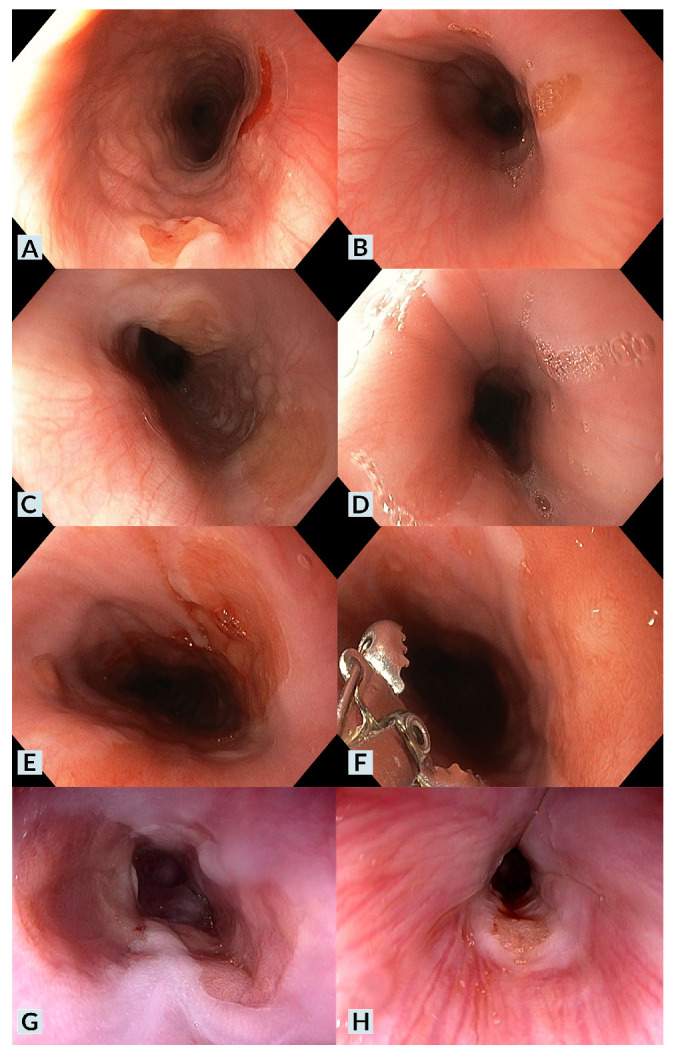
Representative endoscopic features of inlet patches. (**A**): Case 4; a small-sized inlet patch (IP) is observed at the 6 o’clock position. (**B**): Case 8; a small-sized IP is seen at the 2–3 o’clock position. (**C**): Case 2; two medium-sized IPs are observed at the 12 and 4 o’clock positions, respectively. (**D**): Case 5; a small-to-medium-sized IP is seen at the 7 o’clock position. (**E**,**F**): Case 6; two medium-to-large IPs are observed at the 2–3 and 6–7 o’clock positions. Upon close inspection (**F**), a villous/nodular pattern is evident. (**G**): Case 1; two large IPs are seen at the 3–5 and 7–11 o’clock positions, with extension into the mid-esophagus. A clear villous/nodular pattern is visible in both lesions. (**H**): Case 3; a small IP is seen at the 6 o’clock position, showing a villous/nodular pattern along with macroscopic changes in the surrounding esophageal mucosa.

Cervical esophageal symptoms were identified in six cases (67%). The most common symptoms were chronic dysphagia (60%) and GER-related symptoms (60%). Two of these six patients (40%) had experienced at least one prior episode of food impaction. When considering symptomatic IP, those in which there were no gastrointestinal comorbidities or those in which cervical esophageal symptoms were present despite the absence of underlying disease activity (e.g., cases where IP was diagnosed during an UGIE follow-up for EoE with biopsies showing no active EoE), we identified three patients (33%) with symptoms potentially attributable to IP (Case 1, Case 8, Case 9). In two of these cases, the predominant symptom was dysphagia, while in the other case, it was GER-related symptoms. IP symptoms (subacute dysphagia) in the absence of other gastrointestinal comorbidities were identified in only one case (20%) [12]. Notably, this patient had an unusually extensive IP, covering a large portion of the esophageal circumference (Figure 1, Case 1).

In all cases, a distinct lesion was identified at the level of the cervical esophagus, just below the upper esophageal sphincter. The lesion appeared as a well-demarcated, salmon-red plaque (Figure 1). Lesion size and the percentage of the affected esophageal circumference varied, with multiple IP lesions observed in three cases (Cases 1, 2, 6). A nodular or villous pattern was sometimes present at the center of the plaque (Cases 1, 3, 6). In nearly all cases, the IP exhibited friability upon contact with the endoscope and during biopsy sampling, with a tendency to bleed after endoscopic manipulation. Additionally, endoscopic findings of gastric antritis were identified in two cases (22%), while endoscopic findings suggestive of EoE were observed in four cases (44%).

**Figure 2:**
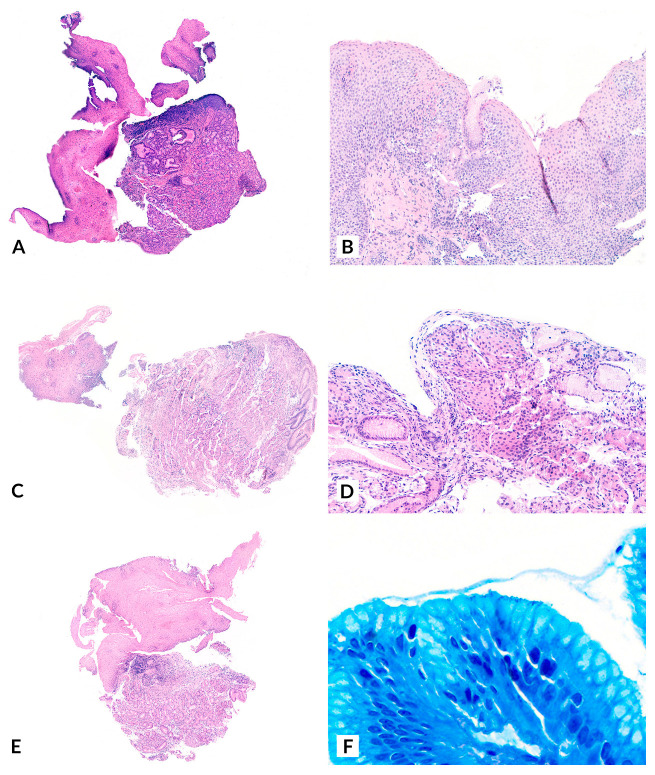
Representative histological features of inlet patches. (**A**): Hematoxylin and Eosin, Case 4; panoramic view showing stratified squamous esophageal epithelium in continuity with gastric-type mucosa and moderate-to-severe chronic inflammation in the lamina propria. (**B**): Hematoxylin and Eosin, Case 7; detail of esophageal mucosa (stratified squamous epithelium) with the presence of gastric foveolar glands and mild chronic inflammation in the lamina propria. (**C**): Hematoxylin and Eosin, Case 9; panoramic view showing stratified squamous esophageal epithelium in continuity with gastric-type mucosa and moderate-to-severe chronic inflammation in the lamina propria. (**D**): Hematoxylin and Eosin, Case 3; presence of specialized gastric epithelium (oxyntic and foveolar) along with stratified squamous esophageal epithelium with mild chronic inflammation of the lamina propria. (**E**): Hematoxylin and Eosin, Case 8; panoramic view showing stratified squamous esophageal epithelium in continuity with gastric-type mucosa and mild chronic inflammation in the lamina propria. (**F**): Giemsa stain, Case 6; presence of bacillary bacterial microorganisms compatible with *H. pylori*.

Histopathological analysis of all cases confirmed gastric heterotopia in the biopsied lesions, establishing the diagnosis of IP (Figure 2). The proportion of specialized gastric epithelium (with oxyntic glands) and non-specialized gastric epithelium (with mucinous glands) varied across biopsies. Regarding the degree of inflammation, all cases exhibited different levels of chronic inflammation. Chronic inflammation was classified as “mild” in five cases (56%) and “moderate-to-severe” in four cases (44%). Among the latter, *H. pylori* was identified in gastric biopsies from three patients (75%), with one case (25%) also testing positive for *H. pylori* at the IP site using Giemsa staining. Acute inflammation was not identified histologically in any of the cases. No cytological atypia, dysplasia, or malignancy was observed in the analyzed cases.

Regarding treatment, all patients except one received oral proton pump inhibitors (PPI). PPI therapy was indicated even in asymptomatic cases due to concomitant conditions such as EoE or *H. pylori*-associated gastritis. Patients diagnosed with *H. pylori* gastritis underwent antibiotic treatment with subsequent confirmation of eradication by stool antigen testing. Additionally, three patients received dietary modifications as part of their management. All patients responded favorably to PPI therapy, with symptom improvement or complete resolution of symptoms. None exhibited refractory symptoms or required endoscopic ablation of the IP.

The duration of follow-up varied among patients; however, eight of the nine cases were diagnosed within the past 12 months, while one was diagnosed seven years ago. Table 1 presents the main sociodemographic and clinical characteristics of the patients included in the study.

## 4. Discussion

The present study reports a series of nine pediatric cases of IP, with a detailed description of their clinical, endoscopic, and histological features.

Regarding the prevalence of IP, recent series have reported an endoscopic prevalence rate of 1.4% [1]. However, significant variability exists among reported rates [1,4], with some specialized centers focused on adult esophageal pathology documenting an endoscopic prevalence of up to 14.8% [5]. Although outdated, pediatric autopsy series have reported even higher prevalence rates, reaching 21% [6]. A plausible explanation for this discrepancy is the underdiagnosis of IP in cases where a thorough and targeted evaluation of the cervical esophagus is not performed during UGIE [16]. In this regard, recent meta-analyses have demonstrated that studies in which endoscopists specifically focused on detecting this lesion reported a higher pooled prevalence of IP [7]. The endoscopists’ expertise in this series, who systematically examined the cervical esophageal region in all patients, likely contributed to a higher number of diagnoses in a relatively short period, potentially identifying cases that might have otherwise been missed. Given that this is a relatively unknown endoscopic finding, selectively located in a region that is challenging to explore and not routinely assessed, we believe that many patients with IP remain undiagnosed.

Immunohistochemical studies have demonstrated that IP and Barrett’s esophagus (BE) share a similar expression profile for Alcian blue pH 2.5/PAS [8], high iron diamine/Alcian blue pH 2.5 [8], cytokeratins 7/20 [9], and mucin-secreting glycoproteins (MUC) [9]. This profile differs from that observed in the ectopic gastric mucosa of Meckel’s diverticulum [8] and healthy antral mucosa [9]. These findings suggest that both IP and BE may originate from submucosal esophageal glands, which are particularly abundant at both the upper and lower ends of the esophagus [9]. Based on this hypothesis, one proposed etiopathogenic mechanism for IP is a focal upper esophageal mucosal developmental anomaly [9]. This theory may also be relevant to the malignant potential of IP through the classic BE metaplasia-dysplasia-carcinoma sequence, as cases of adenocarcinoma arising from IP have been reported [17,18,19,20,21,22,23]. Further supporting this hypothesis, some studies have documented cases of synchronous adenocarcinoma arising from cervical IP and BE-related dysplasia [24].

It is also important to note that the definition of BE varies geographically. In Asian studies, BE is often used interchangeably with gastric metaplasia. In contrast, European and Western studies refer specifically to intestinal metaplasia, which carries a higher risk of progression to adenocarcinoma. The fact that Asian studies establish a diagnosis of BE based solely on the presence of columnar epithelium in the esophagus may contribute to an overestimation of BE cases. From an endoscopic perspective, recent studies have reported concomitant BE in up to 17% of patients with IP [25]. To the best of our knowledge, malignant transformation of IP has not been documented in pediatric patients, likely due to the relatively short pediatric period within a lifetime and the low incidence of BE and esophageal neoplasms in childhood. However, given the unclear malignant potential of IP, this possibility should not be disregarded. Further studies are required to determine whether follow-up protocols for IP are necessary.

In our patient series, a significant proportion had comorbid conditions, including EoE, CD, and Insulin-dependent type 1 diabetes mellitus. However, there is a considerable selection bias, as routine UGIE is not performed in healthy and asymptomatic pediatric patients. Establishing a causal relationship between these conditions and IP is not feasible, and we believe that IP was most likely an incidental finding in patients undergoing UGIE for other indications. The same consideration applies to *H. pylori*-related gastritis, another reason for sometimes performing a UGIE.

Three of our patients exhibited symptoms attributable to IP, while six did not. Determining whether IP solely causes symptoms is challenging, particularly in patients with concomitant conditions, such as EoE, which can present with similar clinical manifestations. The most illustrative case in our series is Case 1, in which the patient had no gastrointestinal comorbidities. In two other cases, gastrointestinal comorbidities were present; however, the absence of EoE activity in the diagnostic UGIE for IP (Case 9) and the lack of other gastrointestinal abnormalities that could justify the symptoms (Case 8) suggest that their cervical esophageal symptoms may be attributed to IP. Nevertheless, it is essential to acknowledge that symptom attribution in these two cases remains challenging.

Some studies have attempted to demonstrate the potential role of IP in symptom development by monitoring acid secretion. In 1985, Jabbari et al. [26] reported a decrease in pH at the IP site following the intravenous administration of pentagastrin, although this was only observed in cases with large IP. Similarly, in 2001, Kim et al. [27] described a patient with pharyngeal symptoms in whom 24 h ambulatory pH monitoring demonstrated acid secretion in the cervical esophagus, with symptom improvement following PPI treatment. In our series, only one patient underwent 24 h pH monitoring, which yielded normal results.

A notable finding in our series was that all IP cases presented some degree of chronic inflammation, with some reaching moderate to severe levels. Interestingly, a potential association was observed between the severity of gastritis in the stomach, the severity of inflammation in the IP, and the presence of *H. pylori*. Specifically, four patients exhibited moderate to severe inflammationin the IP, and in three of them (75%), the same severity was confirmed in the gastric chamber, accompanied by *H. pylori* infection. Additionally, in one of these cases, *H. pylori* was detected within the IP tissue through Giemsa staining. However, the pathological significance of this finding remains unclear.

Several mechanisms could contribute to the development of chronic gastritis in IP, including repeated mechanical trauma due to its location in a high-transit region during swallowing (both saliva and food). It should also be considered that the IP may act as a potential reservoir for *H. pylori* [28,29]. Further studies systematically comparing the degree of gastritis in IP and the gastric chamber are necessary to elucidate this aspect. Similarly, future research should investigate the relationship between IP and *H. pylori*, focusing on prevalence, therapeutic response, and associated symptoms.

Additionally, we identified both types of gastric mucosa within the IP lesions. However, we were unable to assess their relative proportions due to limitations, including the small tissue samples obtained from endoscopic biopsies and the potential heterogeneity of epithelial distribution within the IP. We hypothesize that certain lesion areas may predominantly contain oxyntic glands (gastric corpus-type epithelium), while others may be enriched with mucin-secreting glands (gastric antrum-type epithelium). Investigating this further in living patients is highly challenging; however, autopsy studies, which allow for a comprehensive examination of the entire IP lesion, could provide valuable insights.

IP remains an underdiagnosed and poorly characterized entity in pediatric populations. It is likely congenital in origin and, while often asymptomatic, may occasionally be associated with clinical manifestations. In most cases within our series, IP was an incidental finding during UGIE, and most patients had concomitant gastrointestinal conditions. Chronic inflammation was present in all reported cases.

This manuscript has several relevant limitations that warrant discussion: (1) it is a retrospective study, which may have introduced selection bias or resulted in missing relevant clinical data; while we attempted to mitigate this by conducting an exhaustive review of all patients who underwent UGIE during the study period, this limitation cannot be entirely ruled out; (2) it is a single-center study, and therefore the clinical, endoscopic, and histopathological findings may not be generalizable to other populations, as factors such as genetics, environment, or local clinical practices could influence the presentation and are not accounted for; (3) the sample size is small, which precludes formal statistical comparisons between subgroups of interest and limits the strength of inferential analyses. On the other hand, the study also presents notable strengths: (1) the exhaustive endoscopic and histological documentation, which contributes to a thorough and accurate characterization of the reported cases; (2) the novel contribution of correlating the presence of *H. pylori*, the degree of gastritis, and the level of inflammation within the inlet patch, which opens the door to new lines of investigation.

Reporting additional case series with detailed endoscopic and histopathological correlation is essential to further our understanding of the etiopathogenesis, pathophysiology, diagnosis, management, and prognosis of IP.

## Figures and Tables

**Table 1:** Summary of the patients’ main sociodemographic and clinical characteristics.

Patient	Age	Sex	Medical History	Cervical Esophageal Symptoms	IP Diagnosis	UGIE Findings	*H. pylori*	Pathology	Treatment	Clinical Outcome
1	7y	Female	-	Dysphagia ***	Urgent UGIE for dysphagia	IP (multiple lesions, villous/nodular pattern)	No	IP with mild chronic inflammation	PPI	Favorable
2	12y	Male	Prematurity, EoE	Food impaction. Dysphagia	Incidental finding (EoE control)	EoE EREFS 2 (Edema 1, Furrows 1). IP (multiple lesions)	No	IP with mild chronic inflammation. EoE	PPI	Favorable
3	6y	Male	Adenoid and tonsil hypertrophy, adenoidectomy, and tonsillectomy	None	Incidental finding (Urgent, tonsillar bleeding)	Tonsillar postsurgical bleeding. IP (villous/nodular pattern)	No	IP with mild chronic inflammation	-	Favorable
4	14y	Female	Celiac disease	None	Incidental finding (Celiac disease control)	EoE endoscopic findings. Cobblestone gastric pattern. IP	Yes	Duodenum without villous atrophy but with a slight increase in lymphocytes. Moderate-to-severe chronic gastritis (*H. pylori+*). IP with moderate-to-severe chronic inflammation. EoE	Gluten-free diet. PPI. *H. pylori* treatment *	Favorable
5	11y	Male	Epigastric pain secondary to *H. pylori* gastritis *	None	Incidental finding (abdominal pain)	Petechial antral gastritis. IP	Yes	Chronic duodenitis. Moderate-to-severe chronic gastritis. (*H. pylori+*). IP with moderate-to-severe chronic inflammation	PPI *H. pylori* treatment *	Favorable
6	10y	Male	-	GER-related symptoms. Dysphagia	Elective UGIE for dysphagia	Nodular antritis. IP (multiple lesions, villous/nodular pattern)	Yes	Moderate-to-severe chronic gastritis. (*H. pylori+*). IP with moderate-to-severe chronic inflammation. (*H. pylori + in IP*, Giemsa stain)	PPI *H. pylori* treatment **	Favorable
7	13y	Male	EoE	Food impaction. Dysphagia	Incidental finding (EoE control)	EoE endoscopic findings. IP	No	IP with mild chronic inflammation. EoE	PPI. Topical corticosteroids	Favorable
8	13y	Male	Insulin-dependent type 1 Diabetes Mellitus.Lactose intolerance	GER-related symptoms ***	Incidental finding (abdominal pain)	IP	No	IP with mild chronic inflammation	Dietary modification. PPI	Favorable
9	13y	Male	EoE. Positive serology and compatible genetics (DQ2/DQ8) for celiac disease	GER-related symptoms. Dysphagia ***	Elective UGIE for dysphagia	Duodenal biopsy MARSH 1. EoE endoscopic findings. IP.	No	IP with moderate-to-severe chronic inflammation.	Gluten-free and cow’s milk protein-free diet. PPI + cinitapride.	Favorable

y: years; IP: Inlet Patch; EoE: Eosinophilic esophagitis; PPI: Proton pump inhibitors; GER: Gastroesophageal reflux; UGIE: Upper gastrointestinal endoscopy; EREFS: Eosinophilic Esophagitis Endoscopic Reference Score. *: Treatment with bismuth potassium subcitrate, metronidazole, and tetracycline. Confirmation of eradication after completing the treatment; **: Treatment with omeprazole, amoxicillin, and metronidazole. Confirmation of eradication after completing the treatment; ***: Attributable to the IP either due to the absence of gastrointestinal comorbidities or the lack of underlying gastrointestinal disease activity at the time of IP diagnosis (e.g., controlled EoE after treatment, with biopsies showing no alterations consistent with EoE at the time of IP diagnosis).

## Data Availability

The data used to carry out this study are available upon reasonable request from the corresponding author.
